# Gender-Affirming Phalloplasty: A Comprehensive Review

**DOI:** 10.3390/jcm13195972

**Published:** 2024-10-08

**Authors:** Brandon Alba, Ian T. Nolan, Brielle Weinstein, Elizabeth O’Neill, Annie Fritsch, Kristin M. Jacobs, Loren Schechter

**Affiliations:** 1 Division of Plastic & Reconstructive Surgery, Rush University Medical Center, Chicago, IL 60612, USA; ian_t_nolan@rush.edu (I.T.N.); elizabeth_s_oneill@rush.edu (E.O.); annie_m_fritsch@rush.edu (A.F.); kristin_jacobs@rush.edu (K.M.J.); loren_schechter@rush.edu (L.S.); 2 Department of Plastic Surgery, University of South Florida, Tampa, FL 33606, USA; brielle7@usf.edu

**Keywords:** gender affirmation, phalloplasty, gender affirmation surgery

## Abstract

The goals of gender-affirming phalloplasty typically include an aesthetic phallus and scrotum, standing micturition, and/or penetrative intercourse. Phalloplasty can be performed using both free and pedicled flaps. Complications include flap-related healing compromise and urethral issues, including stricture and fistula. Phalloplasty has high patient satisfaction and has demonstrated improvement in quality of life.

## 1. History of Phalloplasty

The first recorded successful phalloplasty was performed in 1936 by Russian surgeon Nikolaj Bogoraz [1]. The procedure was performed on a 23-year-old male following traumatic amputation using a tubed abdominal flap and a rib cartilage graft for rigidity. Two weeks after flap elevation, the pedicle was divided and the flap was inset. A tubularized flap of scrotal skin was later used for urethral reconstruction. In his lifetime, Bogoraz performed 30 phalloplasties for a variety of indications including trauma, infection, cancer, and congenital defects.

In 1946, Sir Harold Gilles performed the first successful phalloplasty for a transgender man. This reconstructive effort entailed 13 operations over the course of four years. Gilles reconstructed the urethra using abdominal skin, followed by another abdominal flap wrapped around the urethra to reconstruct the shaft. This technique was utilized for the next four decades until advances in microsurgery allowed for more sophisticated free tissue transfer procedures [2,3].

In 1984, Chang and Hwang pioneered radial forearm free flap (RFFF) phalloplasty using a “tube-within-a-tube” technique to reconstruct both the shaft and the urethra in a single stage [3]. The RFFF utilizes the pliable tissue of the forearm supplied by the radial artery. RFFF phalloplasty was later refined by Gottlieb and Levine in 1993, who redesigned the flap, with the urethra placed more centrally in order to optimize flap perfusion [4]. RFFF phalloplasty remains the most common method of phalloplasty to this day [5].

## 2. Indications and Preoperative Evaluation

The World Professional Association for Transgender Health (WPATH) published clinical guidelines, referred to as the Standards of Care (SOC), to provide evidence-based recommendations for the care of transgender and gender-diverse patients [6]. WPATH released Version 8 of the SOC in 2022 [6].

The preoperative process is patient-centered and follows a shared decision-making approach, including a preoperative assessment. Modifiable risk factors should be optimized prior to surgery. Patients are required to discontinue all nicotine products six weeks prior to and after phalloplasty. If patients smoke marijuana, this should be converted to edible forms. Patients should be stable on their current hormonal treatment regimen prior to surgery. Testosterone is typically required in order to achieve hypertrophy of the local tissue. Preoperative hair removal, whether by laser or electrolysis, is often needed at the flap donor site to avoid hair growth within the neourethra.

Prior to surgery, an aftercare plan is developed. Specific postoperative needs include hygiene/self-care, activity restrictions, medications, catheter care, and/or incision/wound management (including the donor site). Patients are educated on local care, activity restrictions, positioning of the flap, and/or changes in their urinary stream.

Many patients travel for gender-affirming surgery. A postoperative plan, addressing travel needs and including follow-up with the patient’s primary team, should be discussed prior to surgery.

## 3. Anatomy

### 3.1. Penile and Scrotal Anatomy

There are three distinct anatomical segments to consider when discussing penile anatomy. These segments are the root, the body, and the glans of the penis. The suspensory and fundiform ligaments suspend the root of the penis from the pubic symphysis [7]. The penile erectile tissue consists of paired corpora cavernosa and a midline corpora spongiosum. The underlying connective tissue at the level of the body of the penis has relatively loose attachments; this allows the skin of the penile shaft to be relatively mobile [8]. In contrast to the loose connective tissue on the body of the penis, the skin of the glans of the penis is less mobile owing to firm attachments within the connective tissue. The external pudendal arteries supply blood to the skin of the penis, whereas the branches of the internal pudendal arteries are responsible for the blood supply of the erectile tissues [7]. The scrotum receives its blood supply from branches of both external and internal pudendal arteries as well as the cremaster arteries [8].

### 3.2. Penile Urethral Anatomy

The penile urethra ranges in length from 18 to 20 cm and, like the penile anatomy, the urethra can be divided into distinct anatomic segments. The most proximal segment of the urethra is the prostatic urethra, which consists of a 3–4 cm section running from the urinary bladder and traveling through the prostate. At the midpoint of the prostatic urethra, there is a 35° turn that is known as the “urethral angle”. When the urethra exits the prostate, it becomes the membranous urethra, which travels 2–2.5 cm from the apex of the prostate to the root of the penis. The bulbous urethra begins after the membranous urethra terminates just distal to the urogenital diaphragm. The bulbous urethra is a 4 cm urethral segment that is found within the corpus spongiosum. Both Littre’s and Cowper’s glands can be found within the periphery at the level of the bulbous urethra. The glands and bulbous urethra play a significant role during erection as the glands release their contents into the urethra when the surrounding muscles contract [9]. The penile urethra is the final segment and is immediately distal to the bulbous urethra. It can be found within the corpus spongiosum, with its distal portion terminating at the level of the external urethral meatus within the glans.

When considering urethral anatomy in the context of phalloplasty, the urethra is often divided into segments. The pars fixa refers to the urethral segments, including the proximal anastomosis and native urethra. The penile urethra continues distally from the pars fixa and is referred to as the pars pendulans [10].

### 3.3. Vulvar, Vaginal, and Perineal Anatomy

The female external genital anatomy is also divided into distinct segments: the mons pubis, labia majora, labia minora, vestibule, and clitoris [7]. Additionally, the clitoris itself can be divided into anatomical segments (crus, root, body, and glans), which are homologous to the penile anatomy. The crura secure two corpora cavernosa to the ischiopubic rami. The corpora cavernosa make up the body of the clitoris. Distal to the corpora cavernosa is a bulb of erectile tissue, which makes up the clitoral glans. The superficial and deep pudendal arteries are responsible for the blood supply to the vulva [7].

### 3.4. Urethral Anatomy

The proximal portion of the urethra begins at the urinary bladder and extends for approximately 4 cm to the anterior vaginal wall. The distal portion of the urethra terminates at the external urethral meatus. There are paraurethral glands, which can be located along the entire length of the urethra. These glands drain their contents at the distal end of the urethra near the meatus. The anterior suspensory ligament of the clitoris and the posterior pubourethral ligament make up the pubourethral ligament. This structure secures and suspends the urethra. The urethra receives blood supply from the pudendal artery [9].

### 3.5. Genital Innervation

The ilioinguinal nerve innervates the anterior third of the labia majora, while the two dorsal thirds of the vulva receive nerve supply from the perineal branches of the pudendal nerve. The lateral vulva is also innervated by the perineal branches. The clitoris is innervated by branches of the pudendal nerve. The clitoral branches are the posterior labial nerves and the dorsal nerve [7,11].

## 4. Surgical Technique

Phalloplasty is a multi-stage process in which an array of free or pedicled flaps can be used for the reconstruction of the phallus and urethra, if desired. The choice of flap involves multiple considerations including patient preference, tissue availability, and donor site morbidity [12]. Generally, the most commonly used flaps are the RFFF, anterolateral thigh (ALT) flap (either free or pedicled), and abdominal-based flaps. The latissimus dorsi, parascapular, and osteocutaneous fibula flaps are less commonly used, but can be useful in select circumstances [13,14]. The advantages and disadvantages of flaps used for phalloplasty are summarized in Table 1.

### 4.1. Staging

**Figure 1:**
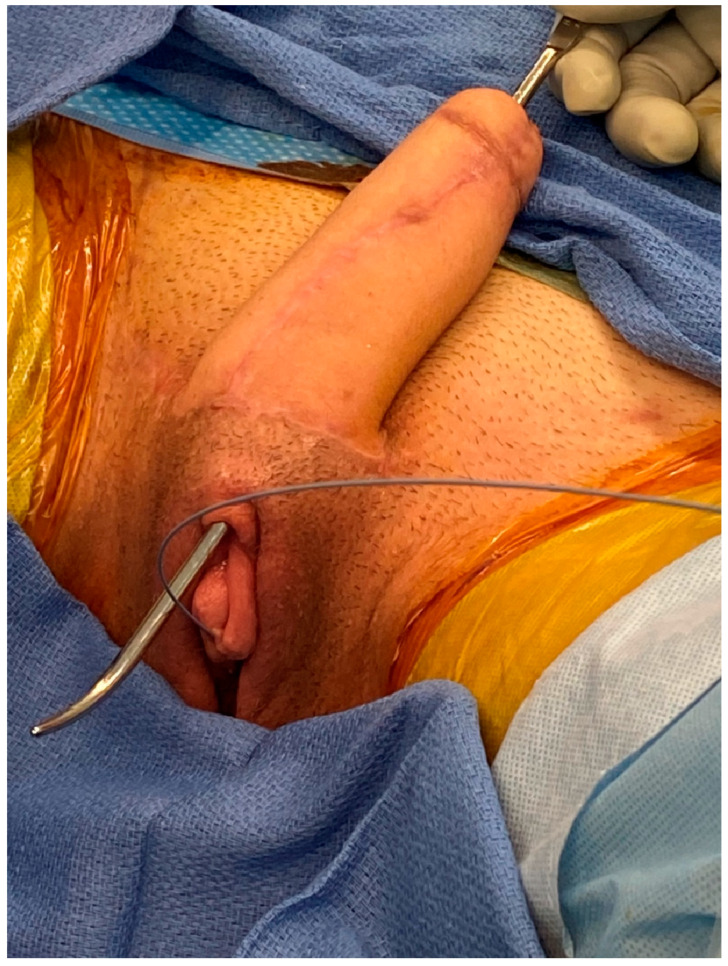
Demonstration of a patient after metoidioplasty (stage I phalloplasty) and radial forearm phalloplasty (stage II); the patient is now presenting for stage III uretheroplasty.

The staging process of phalloplasty can vary between each individual patient, as it is influenced by both surgeon preference and the patient’s surgical goals. Phalloplasty, including the placement of erectile prostheses, is performed in several stages (often three to four separate procedures), largely determined by urethral reconstruction. The pars pendulans, or penile urethra, can be reconstructed at the time of flap phalloplasty utilizing the tube-within-a-tube concept. The pars fixa, or perineal urethra, can be lengthened in a preliminary procedure, followed later by pars pendulans. The lateral (outer) aspect of the labia minora is de-epithelialized. This provides non-hair-bearing, distensible tissue to create the urethra. The labia majora are used to construct the scrotum. Regardless of sequence, many surgeons create the pars pendulans and pars fixa independently, with a subsequent third procedure for anastomosis between the two (Figure 1).

**Figure 2:**
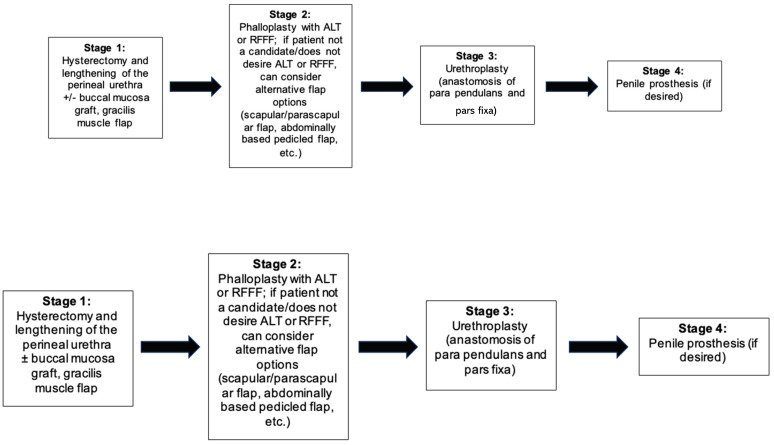
The senior author’s algorithm for masculinizing genital reconstruction for patients who desire urethral lengthening.

Staging of the urethral reconstruction may reduce the risk of urethral complications and the duration of catheter use or urinary diversion [15]. Figure 2 illustrates the senior author’s algorithm for masculinizing genital reconstruction for patients who desire urethral lengthening. For patients who do not desire urethral lengthening, the RFFF, ALT, scapula/parascapular, musculocutaneous latissimus dorsi, or suprapubic flaps may be utilized. For weight loss patients, a pedicled abdominal-based flap may offer the advantage of removing excess lower abdominal tissue. The advantages and disadvantages of each flap option are discussed later in this chapter.

### 4.2. Tube-Within-a-Tube Concept

The RFFF is designed in a tube-within-a-tube fashion with inner and outer vascularized tubes. The thin, pliable tissue of the RFFF is ideal for this technique. The dual tubes also allow for the later placement of a penile prosthesis [16]. The urethra is typically designed to be 16–18 cm in length but ultimately depends on the length of the shaft (as determined by the available length of the RFFF) and the length needed to reach the pars fixa. The urethra is generally designed to be 3.5–4 cm in circumference.

### 4.3. Microsurgical Technique

When performing free tissue transfer for phalloplasty, there are several options for recipient vessels, including the deep inferior epigastric system and the femoral system. The deep inferior epigastric system arises from the external iliac system, superior to the inguinal ligament, and is accessed through a groin incision. There is usually a good size match between the inferior epigastric and radial vessels. An end-to-side arterial anastomosis can also be performed on the superficial femoral artery. If necessary, an arteriovenous transposition between the superficial femoral artery and the saphenous vein can be utilized [17].

### 4.4. Flap Neurotization

Phalloplasty flaps can be neurotized to provide protective and/or erogenous sensation. Techniques for neurotization can vary by recipient nerve, donor nerve, and neurorrhaphy technique. Protective sensation regenerates over the course of 9 to 12 months, after which, a penile prosthesis can be placed. Erogenous sensation of the neophallus is more variable. Oftentimes, patients undergoing phalloplasty are able to achieve orgasm through stimulation of the native clitoris (often buried at the base of the phallus) rather than shaft stimulation. Sensory nerves from the flap can also be coapted to the dorsal clitoral nerve. Recipient nerve options include the lateral antebrachial cutaneous nerve and/or medial antebrachial cutaneous nerves in the RFFF or the lateral femoral cutaneous nerve in the ALT flap. To provide tactile sensation, recipient nerves within the flap can be coapted to the ilioinguinal or iliohypogastric nerves. These nerves can be dissected through an inguinal incision and identified deep to the fascia of the external oblique; they can then be tunneled to the flap for coaptation to the flap nerves [18].

### 4.5. Hair Removal

Prior to phalloplasty, hair removal is often necessary. Hair removal focuses on the portion of the flap that forms the neourethra (volar aspect of the RFF and lateral thigh for ALT). Intraurethral hair may predispose patients to urinary tract infections, fistula, and stones. Electrolysis or laser hair removal may be employed.

### 4.6. Flaps Options for Phalloplasty

#### 4.6.1. Radial Forearm Free Flap

**Figure 3:**
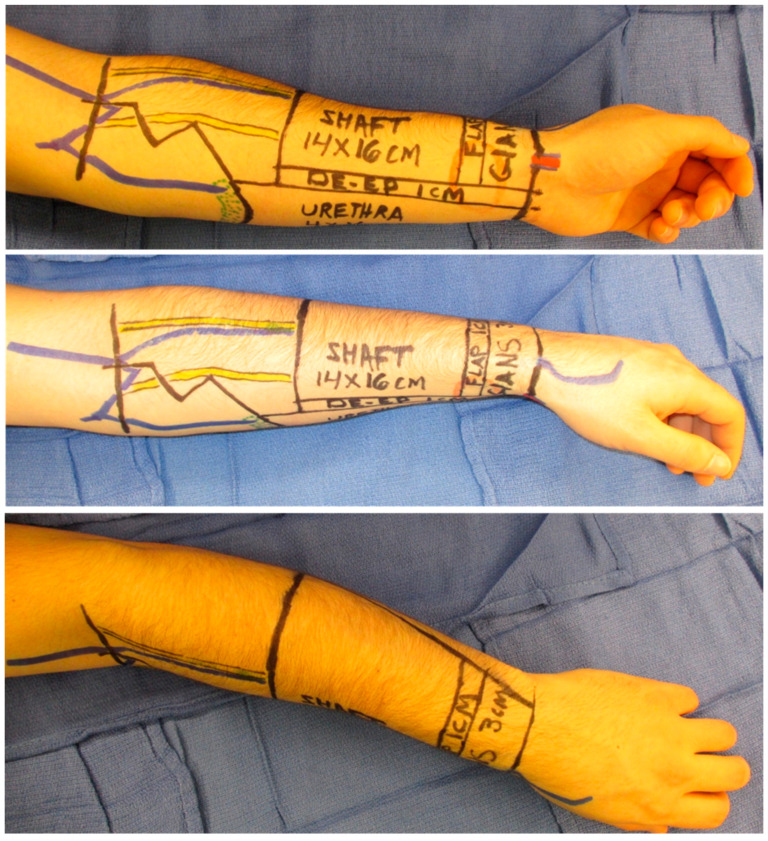
Preoperative markings for radial forearm free flap phalloplasty.

The RFFF is harvested with the radial artery and its vena comitantes (Figure 3).

Oftentimes, a second superficial vein can be found along the ulnar border of the flap and harvested to augment venous drainage of the urethral (i.e., “inner tube”) portion of the flap. Traditionally, the ulnar aspect of the flap is used to construct the urethra and the radial aspect forms the shaft (i.e., “outer tube”).

**Figure 4:**
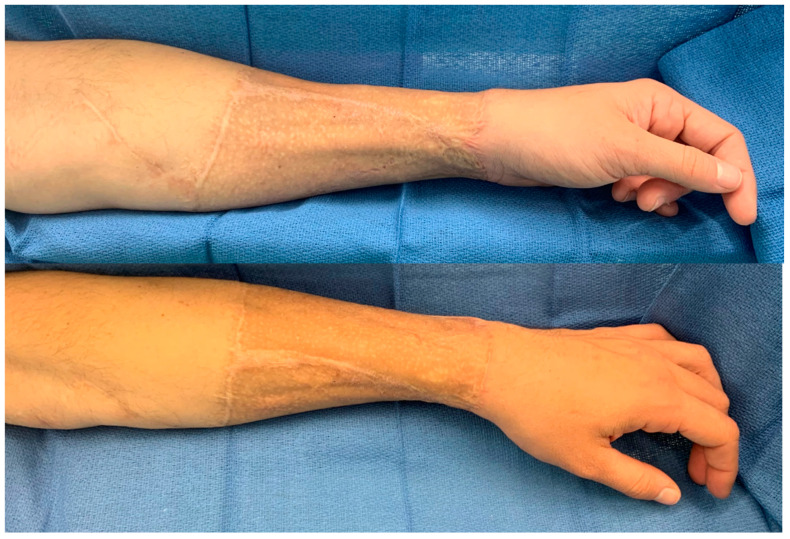
Postoperative donor site from a radial forearm free flap phalloplasty. A bilayer wound matrix was placed immediately after flap harvest followed by split-thickness skin grafting.

While commonly used, the RFFF has drawbacks. For patients with a thin body habitus, the RFFF may not provide sufficient girth. The RFFF results in a conspicuous donor site defect, and hand edema may occur [19]. The senior author’s preferred technique utilizes a bilayer wound matrix (Integra LifeSciences, Princeton, NJ, USA) and negative pressure wound therapy (V.A.C. 3M, St. Paul, MN, USA) on the forearm donor site. Patients begin occupational therapy on postoperative day one. A split-thickness skin graft is placed three weeks later and a compression sleeve is utilized (Figure 4).

**Figure 5:**
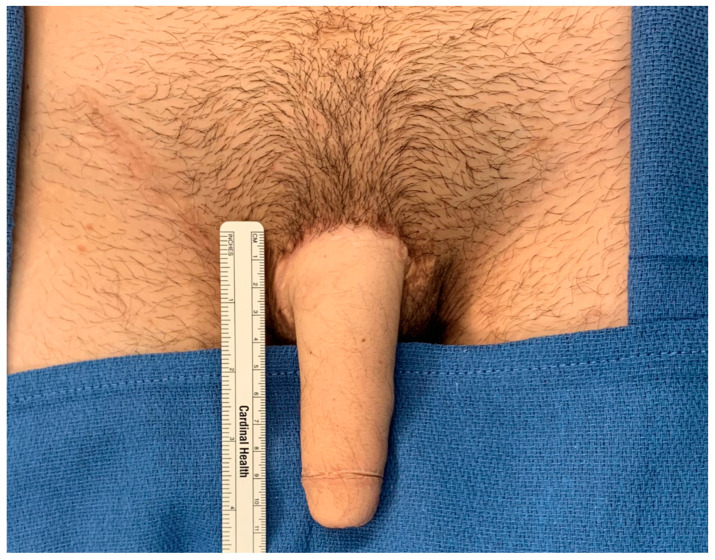
Postoperative result of a radial forearm free flap phalloplasty.

Prior to phalloplasty, hair removal (whether laser or electrolysis) is performed on the ulnar portion of the flap if needed [20]. Figure 5 demonstrates the postoperative result of an RFFF phalloplasty.

#### 4.6.2. Anterolateral Thigh Flap

**Figure 6:**
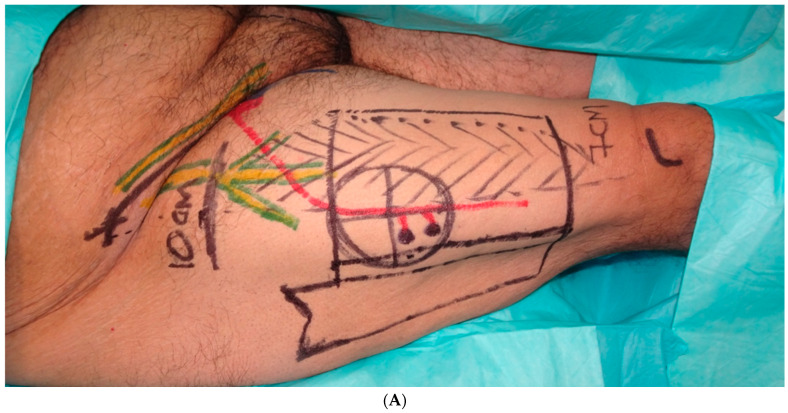

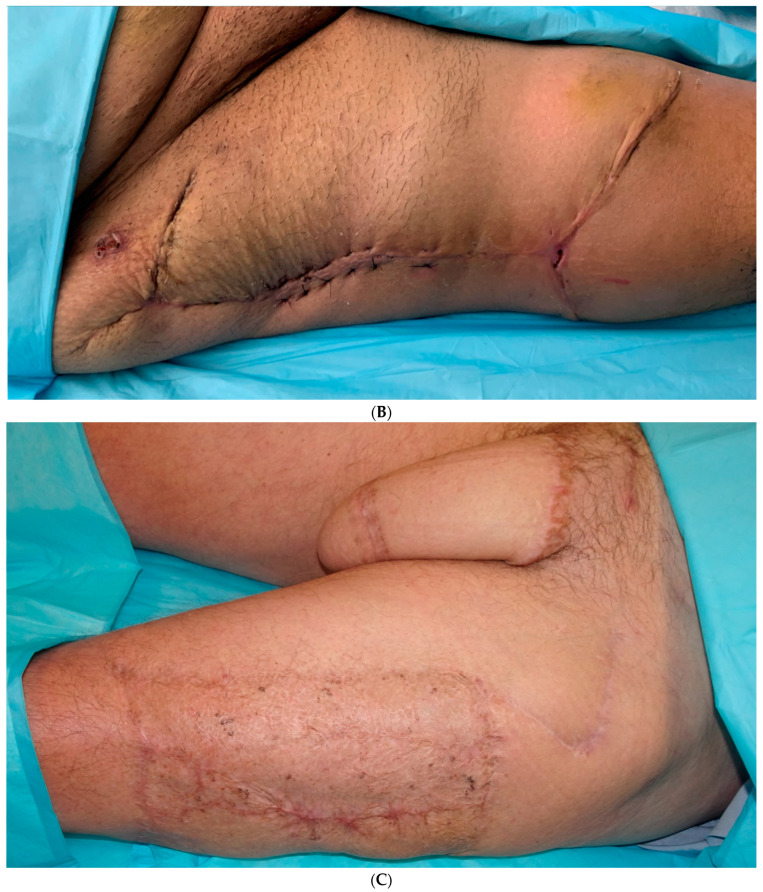
(**A**). Preoperative markings for an anterolateral thigh (ALT) flap phalloplasty. (**B**). Thigh donor site after flap harvest. (**C**). Postoperative result of an ALT phalloplasty and the healed donor site.

The ALT can be used as either a free or pedicled flap (Figure 6).

The flap is supplied by the descending branch of the lateral circumflex femoral artery and innervated by the lateral femoral cutaneous nerve. When used as a pedicled flap, the flap is based on distal perforators to allow for a longer vascular pedicle. The flap is tunneled beneath the sartorius and rectus femoris muscles to facilitate inset. The arterial branch to the rectus muscle may be divided to increase pedicle length. Preoperative CT angiography can be useful in determining the laterality of the flap donor site.

The ALT flap can be relatively large, which affords some flexibility in flap design. Large flap size, however, can also be a primary limitation of the ALT. For some patients, the thickness of the ALT may preclude the “double tube” technique used to create the neourethra [21]. Generally, a flap thickness of less than 1.5 cm is preferred. For large flaps in which a double tube design is not possible, secondary skin grafts or flaps can be used.

### 4.7. Abdominal-Based Flaps

Pedicled flaps from the lower abdomen can also be used for phalloplasty. In this technique, two mirrored, cutaneous flaps based on the superficial arteries and veins of the lower abdomen are approximated at the midline. These flaps can be ideal for patients in which urethral reconstruction is not desired, as these flaps are not amenable to the “tube-within-a-tube” design. Abdominal-based flaps are also useful for patients who are not good candidates for RFFF or ALT phalloplasty. Donor site morbidity and operative time are relatively low compared to other free flap donor site options.

### 4.8. Back-Based Donor Sites

While the forearm and anterolateral thigh are the most common donor sites for phalloplasty, the back is an alternative donor site. The myocutaneous latissimus dorsi (MLD) flap is based on the thoracodorsal system. It includes a small portion of the latissimus dorsi muscle. Neurotization may be performed between the thoracodorsal nerve and the ilioinguinal or other regional recipient site nerves, although sensory outcomes of the phallic shaft are poor [22].

Benefits of the latissimus dorsi flap phalloplasty include a reliable flap with ample tissue. Proponents of the technique argue that the presence of latissimus dorsi muscle tissue may decrease the rate of flap contracture when compared to fasciocutaneous flaps, which may ultimately lead to a larger phalloplasty. The increased size of these flaps generally accommodates the placement of an erectile prosthesis. Additionally, the scar is well hidden underneath clothing.

Downsides of the MLD include relatively thick tissue in the upper lateral back, which may result in a phallus that is too large for many patients’ aesthetic or functional goals. Especially in obese patients, this flap may not be a viable option. Additionally, urethral reconstruction is typically performed in a staged fashion. Even in thin patients, the back tissue often requires pretreatment with massage therapy to allow for a pliable flap that can be effectively tubularized [22].

While neurotization has been described, sensory outcomes for the latissimus dorsi flap have been very limited. The thoracodorsal nerve is primarily a motor nerve and may not carry adequate sensory input to the flap [23]. Lack of sensation predisposes patients to soft tissue injury and limits the ability to place and retain an erectile prosthesis.

Another donor site option from the back is the scapular or parascapular flap [24]. They are both axial flaps, based on the transverse or descending branch of the circumflex scapular artery, respectively. Similar to the myocutaneous latissimus dorsi flap, these flaps have the advantage of a concealed donor site scar. However, the flaps lack a sensory nerve.

### 4.9. Metoidioplasty

Metoidioplasty constructs a phallus using the hormonally-enlarged clitoris [25]. The resulting phallus is approximately 5–9 cm in length, which typically allows for standing micturition but not for penetrative intercourse. Some patients choose metoidioplasty over phalloplasty as it is generally a less complex procedure with less donor site morbidity. Metoidioplasty also maintains erogenous sensation of the existing glans. A metoidioplasty can be used as the first stage of a “meta first” phalloplasty, in which the perineal urethra is reconstructed at the time of the metoidioplasty.

### 4.10. Adjunct Procedures

A glansplasty reconstructs the coronal ridge. In this procedure, a distal-based partial thickness flap is elevated and folded upon itself (i.e., the Norfolk technique) [17]. Glansplasty may be performed at the time of RFF phalloplasty or as a secondary procedure. When an ALT flap is used, the glans are created secondarily.

Penile prostheses can be implanted to achieve rigidity and allow for penetrative intercourse [26]. The devices are either malleable or inflatable (hydraulic). Inflatable prostheses include a fluid reservoir buried in the lower abdomen or scrotum and a pump, typically placed in the hemiscrotum. Testicular prostheses can also be placed. Oftentimes, a pump for a penile prosthetic is placed on one side of the scrotum, and a testicular implant is placed in the contralateral scrotum. The choice of malleable versus inflatable prosthesis is based on patient preference and flap dimensions (tissue quality and volume). Malleable prostheses have higher rates of erosion and tend to be better tolerated in larger flaps or shaft-only flaps. For RFFFs, single-cylinder inflatable prostheses may be preferable. These prostheses are not approved by the United States Federal Drug Administration (FDA) for use in gender-affirming phalloplasty and their use in these procedures is considered off-label. One specific implant, the ZSI-475 FTM (Zephyr Surgical Implants, Geneva, Switzerland), has been available in European markets since 2016 and is purportedly designed specifically for gender-affirming phalloplasty [27]. Studies on erectile prostheses in gender-affirming phalloplasty are heterogeneous, with significant variability in implant type, operative technique, and outcome measures.

## 5. Outcomes

### 5.1. Patient-Reported Outcomes

Wang et al. published a systematic review on gender-affirming phalloplasty outcomes in 2022 [28]. The authors found that most patients (529 of 574 patients, 92.2%) were able to achieve standing micturition. A majority of patients had tactile sensation of the neophallus (771 or 821 patients, 93.9%). The systematic review reported that 59.9% of patients had erogenous sensation or “sexual function” (160 of 267 patients, 59.9%); “sexual function” was typically defined as the ability to achieve penetrative intercourse. Only one paper reported on patients’ ability to orgasm; Monstrey et al. reported that all 280 patients in their cohort of RFFF phalloplasties were able to achieve orgasm postoperatively [29].

A handful of studies have also reported on aesthetic/cosmetic outcomes after phalloplasty. Garaffa et al. found 97.4% of patients (112 of 115 patients) undergoing RFFF to be satisfied with their postoperative phallic size and cosmesis [30]. In a cohort of 24 patients undergoing abdominal-based phalloplasty, Terrier et al. found 95% of patients to be satisfied with appearance, 81% to be satisfied with length, and 71% to be satisfied with circumference [31]. In looking at overall quality of life, Papadopulos found that 91% of patients would undergo surgery again, 84% would recommend the surgery to others, and 81% had improved quality of life [32].

### 5.2. Complications

Preoperative counseling helps to establish realistic expectations and possible complications. The type and incidence of complications vary between flaps, urethral reconstruction techniques, and the use of prostheses.

In a series of 229 patients undergoing RFFF phalloplasties, Wirthmann et al. reported that 16.8% of patients experienced delayed wound healing, defined as wounds open for more than 21 days postoperatively; 13.8% of patients had delayed wound healing that required operative intervention [33]. Other studies have reported wound healing complications between 0% and 12.5% [34,35]. Total flap loss is uncommon and is estimated to be around 3% [28]. Partial flap loss is more common, occurring in about 11% of cases [28]. The incidence of flap loss is relatively consistent among different flaps (e.g., RFFF vs. ALT) [28]. Reported rates of surgical site infection are highly variable and range from 0.0% to 41.7% [31,36]. Reported hematoma ranges range from 0.0% to 22.7% [35,37].

The neourethra is particularly prone to stricture and fistula. In a systematic review, Wang et al. found a urinary fistula rate of 34.1% and a stricture rate of 25.4% [28]. While strictures and fistulae can occur at any aspect of the neourethra, they are most common at the anastomoses [15]. To mitigate these risks, several authors have reported on this use of flaps to bolster the urethral anastomoses, such as a pedicled gracilis muscle flap [38]. Whether urethral reconstruction should occur in a single- or multi-stage process remains a topic of debate [39,40].

Implant-related complications are common. Oftentimes, these complications ultimately necessitate explantation. Overall five-year device survival has been estimated at about 75% [27]. Infection rates range from 4.2% to 40.0% [41,42] and other complications include mechanical failure (15.4%), malposition (4.7%), and chronic pain [43,44,45]; it is estimated that “pain” is the cause of up to 5% of device failures [46].

## 6. Conclusions

Gender-affirming phalloplasty is a complex reconstructive effort, and a variety of flaps and techniques are utilized. The surgeon should consider each patient’s goals and preferences when collectively determining a surgical plan.

## Figures and Tables

**Table 1:** Overview of flaps for phalloplasty.

Flap	Advantages	Disadvantages
Anterolateral thigh	-Relatively large -Can be used as free or pedicled flap -Minimal donor site functional morbidity	-Can be excessively bulky
Radial forearm	-Thin, pliable tissue	-Donor site cosmetic and functional morbidity -May not provide sufficient girth for neophallus
Abdominal-based flap	-Shorter operative time than that for free flaps -Relatively low donor site morbidity	-Not ideal for urethral lengthening (not amenable to “tube-within-a-tube”)
Latissimus dorsi	-Thin, pliable tissue	-Donor site functional morbidity

## Data Availability

No new data were created or analyzed in this study. Data sharing is not applicable to this article.
